# When Silence Breaks: The Influence of Pure Tones and White Noises on Conditioned Flight Responses

**DOI:** 10.1002/brb3.70561

**Published:** 2025-05-19

**Authors:** Sebastiano Francesco Matarazzo di Licosa, Andrea Stefano Moro, Mattia Ferro, Antonio Malgaroli, Jacopo Lamanna

**Affiliations:** ^1^ Center for Behavioral Neuroscience and Communication (BNC) Vita‐Salute San Raffaele University Milan Italy; ^2^ Department of Psychology Sigmund Freud University Milan Italy; ^3^ Faculty of Psychology Vita‐Salute San Raffaele University Milan Italy; ^4^ San Raffaele Turro IRCCS Ospedale San Raffaele Milan Italy

**Keywords:** animal behavior, fear conditioning, Pavlovian learning, serial compound stimulus, SCS, white noise, sensory systems

## Abstract

**Background:**

The flight response is part of the repertoire of adaptive behavioral responses all animals possess and use to face threats coming from their environment. Compared to the other responses, flight requires a high degree of physical effort and is thought to be related to those active coping strategies that can be observed in several psychopathological conditions, including anxiety and depressive disorders. In recent years, a new protocol of auditory fear conditioning has been shown to induce a learned flight response in mice, based on a conditioned stimulus that includes pure tones and white noise, the serial compound stimulus (SCS).

**Methods:**

In this review, we examine the effects of stimulus characteristics in fear learning paradigms, particularly in the context of the recently developed SCS paradigm. We will discuss how factors such as conditioned stimulus (CS) modality (e.g., tone versus white noise), stimulus salience, and the temporal relationship between stimuli influence conditioned flight responses.

**Results:**

For the study of both physiological and maladaptive behaviors, fear conditioning still represents the paradigm of choice, e.g., for the modeling of psychiatric conditions such as post‐traumatic stress disorder or phobias. Albeit its relevance in this context, up to now only a few studies have focused on developing procedures for eliciting conditioned flight responses in the laboratory, in favor of freezing/immobilization, the so‐called fright response. The SCS protocol poses new interesting questions on the impact of noises and other stimuli on learning and behavioral responses.

**Conclusion:**

The discovery of SCS already led to interesting findings in the neurobiology of fear learning and shows great potential for the study of maladaptive responses in animal models of psychopathology.

## Graphical abstract

A novel behavioral paradigm by Fadok et al. (2017) pairs a sequence of pure tone and white noise with footshock. After learning, the mouse shows freezing during pure tones and jumps during white noise. This discovery produced shows great potential for the study of fear learning and maladaptive responses.

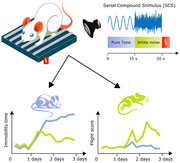

## 1 Introduction

The flight response, also known as the escape response, is part of the repertoire of adaptive responses all animals possess in order to steer clear of threats. Such a phenomenon was first described by W. B. Cannon in 1929 as part of the fight‐or‐flight response and is now widely recognized in the clinical community as well as in the culture at large. Indeed, the flight response is a typical fear‐ and panic‐related behavior and is viewed as a crucial factor in the emergence of psychopathological conditions, such as exaggerated and/or maladaptive defensive responses to perceived threats (Thompson et al. 2014). Extensive research on defensive responses (mainly in translational neuroscience studies) has evidenced how the flight response is innate, species‐specific and contingent upon threat, proximity and context of exposure (Bolles 1970; Borkar and Fadok 2021).

While the study of innate flight strategies has made significant progress in developing paradigms, especially in semi‐naturalistic conditions and in analyzing the trajectories animals take to seek safety (Campagner et al. 2022; Domenici and Ruxton 2015), the investigation of learned flight responses within the context of fear conditioning remains considerably limited. In particular, the significance of conditioned flight lies in its ability to model active coping responses, which are distinctly different from passive coping strategies such as freezing. Differentiating these responses is not only pivotal for advancing our understanding of neuronal circuits involvement, but it also has critical implications for translational research into stress‐related disorders such as PTSD. Thus, the use of a simple, reproducible laboratory paradigm is essential.

However, researchers working in the field of behavioral science know very well how hard it can be to obtain reproducible findings, especially in behavioral paradigms. Since the use of genetically modified mouse models had become widespread in the 90s, it had also become clear that very different results could be obtained within similar paradigms, even when using the same mouse phenotypes (Voikar 2020). Based on this, studies that use carefully controlled and known phenotypes of mutant mice are still vulnerable to threats to validity.

Main factors that became known to hinder an experiment's validity were the impact of the environment and the effect of the experimenters. Another relevant aspect to mention is that different inbred strains of mice may reduce the validity of the results because of their own peculiarities. For example, FVB/N mice often suffer from retinal degeneration, caused by mutations due to inbreeding, thus making them not ideal subjects for spatial navigation tasks (Voikar 2020). Another issue concerning behavioral studies using animal models is that often researchers implement conditional stimuli without knowing what consequences they might have on the behavioral tasks. One of the most widely used paradigms in behavioral research is Pavlovian fear‐conditioning. This approach consists in exposing a subject to a conditioned stimulus (CS, e.g., a pure tone) paired with an unconditioned aversive stimulus (US, most commonly an electrical footshock) and subsequently observing the defensive responses elicited by the unconditional stimulus as well as conditioned responses elicited by the CS following repeated pairings with the US (Maren 2001). Given the central role of stimuli in such paradigms, it is evident that a lack of nuance in considering their properties may compromise experimental reliability.

Reducing the variability of a response as inherently flexible and variable as flight behavior may seem paradoxical. However, this approach is driven by pragmatism rather than dogma: to investigate the underlying neural circuits, it is essential to simplify as much as possible and minimize variability.

Beyond issues of reproducibility in behavioral neuroscience, the study of the flight response is further complicated by theoretical debates regarding its very definition. As posited by the Predatory Imminence Theory, the flight response falls into the category of the so called circa‐strike defenses: explosive, energetic and panic‐like responses, including jumping, biting and vocalizations, all aimed at getting away from the immediate threat (Fanselow and Lester, 2013). Because these defensive behaviors are considered responses to dangerous unconditioned stimuli (e.g., a predator lunging at the animal), some experts argue that flight cannot be induced as a conditioned response. Consequently, until recently, flight responses have been mostly studied as shock‐escape behaviors, i.e., an escape response following a painful unconditioned stimulus (e.g., Franchina 1969; Ritter et al. 2024). However, recent studies using white noise in experimental paradigms have challenged this assumption, demonstrating that flight can be triggered in a conditioned manner (Fadok et al. 2017). These findings facilitate research into the neural mechanisms underlying defensive state transitions and suggest the need to reconsider existing theories on defensive behavior.

In this context, we believe that a deeper understanding of the different properties of the stimuli used in behavioral paradigms is warranted in order to distinguish the innate effects caused by such stimuli from those caused by learning. Accordingly, in this review we will analyze fear conditioning paradigms, in particular Pavlovian fear conditioning, that have been widely used in studying behavioral defensive responses, with the focus on understanding the associations made between environmental stimuli and the enacted fear responses (Bolles 1970; Maren 2001). In particular, we will summarize and discuss the available evidence about the effects of different stimulus characteristics in such behavioral paradigms.

## 2 Serial Compound Stimulus (SCS) Promotes Flight

**FIGURE 1:**
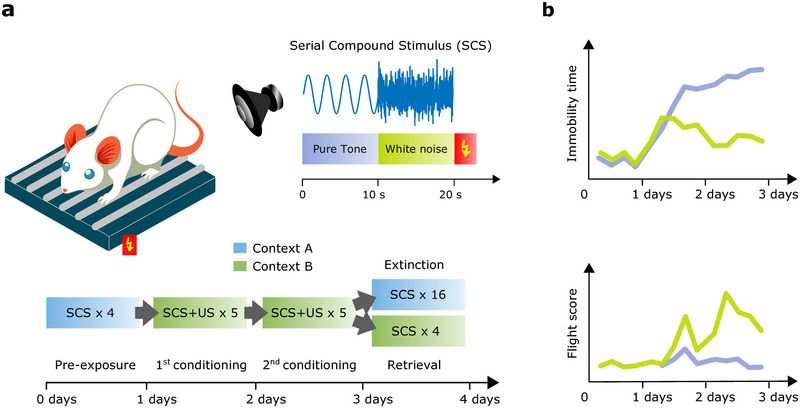
Fear conditioning based on the serial compound stimulus (SCS) evokes flight responses. (a) A novel behavioral paradigm by Fadok and colleagues (2017) pairs a sequence of pure tone (10 s) followed by white noise (10 s) with footshock (↯). (b) After repeated associations (2 days), a complex conditioned anticipatory response at retrieval (day 4): (i) the expected increase of immobility during pure tones (freezing; top graph); (ii) an increase of motor activity during white noise (flight response; bottom graph), characterized by fast jumps (adapted from (Fadok et al. 2017)).

It is noteworthy that studies using aversive CS stimuli can also be employed to investigate various behaviors, such as conditioned inhibition and place aversion. However, the aim of this manuscript is to focus on the contrast between freezing and flight responses, as their opposition provides valuable insights into the neurobiological mechanisms underlying major psychiatric disorders. Indeed, fear conditioning studies have mostly concentrated on reproducing and analyzing fear responses, such as freezing, in order to understand the underlying neural processes of defensive states. Only recently has the attention shifted towards panic responses, chiefly the flight response. In 2017, a study by Fadok et al., which used white noise as an aversive stimulus capable of inducing avoidance behaviors in mice, presented a Pavlovian fear conditioning paradigm specialized in promoting escape behaviors (Fadok et al. 2017). Figure 1 depicts a scheme of the behavioral protocol and of the produced behavioral response (adapted from (Fadok et al. 2017)).

The chosen CS that best suited their experiment was an SCS, a series of sounds made up of pure tones followed by white noise that strongly induced both conditioned freezing and flight in sequence. The conditioning phase of the experiment lasted 2 days, during which the SCS was paired with a 1 second footshock. After this phase, mice showed increased freezing in response to pure tones, and flight during white noise exposures, as shown by an increase in the “flight score” that quantified flight behaviors by measuring increases in speed and number of escape jumps. Further behavioral analyses uncovered that conditioned flight was context‐dependent and was quickly extinguished in extinction trials that only consisted of the SCS without the US (Fadok et al. 2017).

It is worth noting that Fadok and colleagues were not the first to use white noise as the CS in fear conditioning paradigms (Walasek and Zielimski 1989). Previous studies have also employed Pavlovian fear conditioning to analyze escape behaviors (Li and He 2013), and SCS paradigms had already been used for conditioned avoidance studies (Levis and Stampfl 1972). However, what makes Fadok et al.’s findings particularly noteworthy is their demonstration that they successfully induced flight as a conditioned response in a laboratory setting, thanks to their novel fear‐conditioning paradigm. This discovery has attracted significant attention, as it directly challenges prominent and widely accepted theories of defensive behavior, as mentioned above.

Building upon previous findings on the role of the amygdala in expressing conditioned defensive responses (Maren and Quirk 2004), Fadok and colleagues used in vivo optogenetics and extracellular recordings of discrete cell types in mice to understand their role during freezing and flight (Fadok et al. 2017). After identifying specific cell populations of inhibitory GABAergic neurons that express corticotropin‐releasing factor (CRF), somatostatin (SOM), or protein kinase C δ (PKCδ) in the central amygdala (CEA), they found that inhibition of CRF+ neurons eliminated conditioned flight behavior. Additionally, electrophysiological recordings of the CRF+ neurons’ response showed an increase in activity following white noise, as well as increased activation during flight but not during freezing. Recordings of SOM+ neurons in the lateral CEA (CEl) during the flight paradigm revealed a strong inhibition in response to white noise and an increase in firing rates during high levels of contextual freezing (i.e., during the 3‐minute baseline prior to the SCS). Most of the SOM+ neurons that were identified showed activation during freezing and were inhibited during flight, thus hinting at a possible interaction between freezing and flight networks. In accordance with this, exogenous photo‐excitation of CRF+ neurons during pure tones, inhibited passive freezing responses and evoked more active behaviors. Moreover, excitation of SOM+ neurons during white noise decreased flight and increased freezing behavior. Researchers ascribed this mutual exclusion between freezing and flight to a reciprocal inhibitory connection between CRF+ and SOM+ neurons in the CEA network, based on recordings of postsynaptic currents between the two cell populations. Such a circuit would therefore be crucial in enabling rapid switches from more passive to active defensive strategies (e.g., from freezing to flight) (Fadok et al. 2017).

A follow‐up study was done, using the same SCS‐US Pavlovian fear conditioning paradigm mentioned above, in order to investigate possible sex differences in the type and intensity of defensive responses enacted by mice during the experiment (Borkar et al. 2020). The researchers found noteworthy sex differences regarding the freeze response, with it being more prevalent in females. Specifically, during the extinction session, females expressed a significant increase in freezing in response to white noise, and in late extinction sessions they also froze to pure tones. Furthermore, a higher total freezing time was measured in female mice, compared to males on day 3 of the experiment, namely caused by an increase of freezing during white noise exposures. Females also showed markedly enhanced freezing during intertrial intervals (ITI), attributable to high total ITI freezing and high duration of each ITI freezing bout. However, male and female mice did not show any differences in conditioned flight behavior (Borkar et al. 2020).

In the same study, rearing, grooming, and tail rattling were analyzed to elucidate sex differences in the effect of fear conditioning on the mice's overall behavioral expression. Rearing is a typical exploratory behavior that mice display in novel contexts, and it is generally described as the amount of time spent standing on rear limbs to better explore the environment (Laštůvka et al. 2020; van Abeelen 1970). In Borkar and collaborators’ conditioned flight paradigm, both male and female mice showed an initial decrease in rearing behavior, probably associated with stress during conditioning, followed by a slight return during extinction training. Most importantly, however, male mice seemed to rear more than females during the third day of experimentation, possibly showing greater resilience to stress.

Grooming is a behavior that serves both hygiene maintenance and stress reduction, as well as other physiological processes, in mice (Kalueff et al. 2016). The general trend of grooming behavior in stressful environments was described as following an “inverted‐U shape curve”: rarely appearing in low arousal conditions, increasing at moderate levels of arousal and decreasing in high arousal conditions (Borkar et al. 2020). A significant decrease in grooming behaviors was recorded during the conditioning phase in both sexes, and an increase during extinction, with grooming reaching its peak at the last sessions of extinction. Notably, male mice showed more grooming on day 3 in comparison to females, indicating a possible deficit in female stress coping strategies.

Finally, tail rattling is a common aggressive defensive response elicited by threatening situations between conspecifics (John 1973), and in both female and male mice, tail rattling increased in the first day of the conditioning paradigm and decreased during extinction, with the only sex difference being that females showed such a behavior more than males on the first day of conditioning. These findings suggest possible differences in the regulation of defensive and adaptive behaviors between the two sexes, providing greater insight on the underlying sex‐dependent factors that contribute to, for example, a greater female susceptibility in developing anxiety‐related disorders and post‐traumatic stress disorder (Borkar et al. 2020).

## 3 Stimulus Salience Triggers Flight

Since the SCS is composed of a repeating and constant series of auditory stimuli that immediately precede a footshock during the conditioning training, researchers have posited that the defensive behaviors exhibited by mice in response to single components of the SCS could be elicited by learned temporal relationships between the conditioned stimuli and the unconditioned stimulus (Dong et al. 2019). However, a rival hypothesis put forward was that what drives the particular defensive behaviors evoked by the SCS could be the specific properties of the auditory stimuli (white noise and pure tones) (Hersman et al. 2020). This is exactly what Hersman et al. tried to ascertain in their 2020 study. Firstly, they tested whether switching the order of the pure tones (TN) and white noise (WN) within the SCS would reverse the behaviors induced in mice models, in order to test whether the temporal relationships of the stimuli explain the elicited behaviors. They observed an increase in movement during WN compared to TN, regardless of the order of presentation, as well as a higher amount of both freezing and escape‐like behaviors within the group exposed to SCS paired with US, compared to a control group that had a 60 s gap between SCS and the US. Consequently, the data indicated that reversing the stimulus order does not qualitatively alter the behaviors, albeit a few quantitative differences did emerge when WN preceded the TN, eliciting significantly less active defensive behaviors. However, this was possibly due to the fact that SCS, which increase in salience, are perceived as more naturalistic and evoke greater arousal levels. A similar effect was also observed in humans, as tonal stimuli that increase from low to high frequencies tend to be rated as more alarming and are associated with an increase in attention and arousal (Catchpole et al. 2004; Owren and Rendall 2001).

It is important to mention that, in contrast to Hersman and colleagues’ finding regarding the lack of a qualitative switch in behavior following the presentation of a reversed SCS (Hersman et al. 2020), Dong et al. uncovered the exact opposite in their 2019 study (Dong et al. 2019). After conducting the same type of Pavlovian fear conditioning using an SCS followed by a footshock, they reported that using a reversed SCS (i.e., white noise, pure tone then footshock), the behaviors elicited by the animals were also reversed (freezing during white noise and flight during tones) (Dong et al. 2019). Although the factors that caused such different results aren't entirely clear, Hersman et al. (2020) ascribed their contrasting outcome to the use of a different mouse strain that may imply a difference in the underlying neural processes that manage threat assessment. Nevertheless, it is still important to consider the temporal relationships of the stimuli as a key element in SCS paradigms (Borkar and Fadok 2021; Hersman et al. 2020).

Overall, the results from Hersman et al.’s (2020) study seemed to hint towards the idea that intrinsic properties of the stimuli are involved in threat prediction. In accordance with this, the researchers found that white noise is naturally more salient and arousing than 7.5 kHz pure tones in unconditioned mice that had not been previously exposed to these stimuli, based on physiological and behavioral measures, such as greater pupil dilation and locomotor activity.

Given that the differences in the perception and response to TN and WN, attributable to their difference in saliency, seemed to reflect the perception of two stimuli on different points of the threat imminence continuum (Fanselow et al. 1988), Hersman et al. (2020) believed that even the 7.5 kHz tone, at higher sound pressure levels (SPL), would be perceived as more imminent and therefore elicit more flight than the same TN at a lower SPL. An “SPL step test” was planned and performed to test this hypothesis, consisting of an SCS made up of two 7.5 kHz pure tones, one of which remained constant at a fixed SPL (75 dB), while the other began at a lower level (55 dB) and increased by 5 dB each trial, until a maximum of 105 dB. Results showed that at lower SPL (under 85 dB), freezing was the predominant response in mice, whereas flight responses began to emerge as the 7.5 kHz CS surpassed 90 dB. The same “SPL step test” was performed using WN as the CS, showing similar results to the previous TN condition. Thus, both TN and WN were able to elicit freezing and flight depending on the SPL magnitude.

The same study also reported that the frequency of the CS by itself can influence the type of defensive behavior induced. This was demonstrated using a fear conditioning paradigm with an SCS composed of 3 and 12 kHz pure tones. Mice showed greater escape behavior and less freezing in response to the 12 kHz than the 3 kHz, irrespective of the order of presentation during training and at equivalent SPL (Hersman et al. 2020). These experiments raise several intriguing questions. First, it remains to be determined whether the flight response is truly conditionable, given that WN may no longer reliably induce it while a pure tone potentially does. Even more complex is the role of WN, which appears to act more as a facilitator of flight behaviors than as a direct inducer. To gain a deeper understanding of these phenomena, it is essential to investigate the underlying neural correlates.

## 4 The Central Amygdala's Role in Flight Responses

More recent studies, following Fadok et al.’s pioneering 2017 article, uncovered additional data linking the amygdala to context‐dependent flight behavior in the SCS paradigm (Totty et al. 2021). After confirming that a serial‐compound stimulus can induce context‐dependent freezing and flight‐like behaviors in rats (given that prior studies using SCS were conducted on mice), Totty and colleagues hypothesized that by inhibiting brain regions that are vital for processing contextual and/or cued fear (such as the bed nucleus of the stria terminalis and the central amygdala), evoked flight behaviors could be suppressed. After habituation and conditioning training, two experimental groups of rats were created, one of which received saline (SAL), while the other received the GABA_A_ (γ‐aminobutyric acid type A) receptor agonist muscimol (MUS), both of which were administered immediately prior to the retrieval testing by means of a cannula targeting the bed nucleus of the stria terminalis (BNST) or the central amygdala (referred to as the central nucleus of the amygdala in the article). Results showed that following an inhibition of either CEA or BNST (thanks to MUS injections), the baseline freezing levels diminished, pointing at a possible reduction in context‐dependent fear. Rats that received CEA inactivation showed less freezing in response to the pure tones of the SCS, and along with the BNST group, showed lower levels of flight‐like behaviors. Thus, these data seem to indicate that flight responses induced by SCS paradigms can be blocked by inhibiting neural networks that modulate contextual fear, such as the BNST and the CEA (Totty et al. 2021).

Recently, evidence was uncovered regarding the origin of neuronal activation of the CEA, namely a previously unknown projection from the medial prefrontal cortex (mPFC) to the CEA (Borkar et al. 2024). Researchers found a significant number of CEA‐projecting neurons located in the dorsal peduncular nucleus (DP), a specific area of the mPFC linked to behavior elicited by psychosocial stress (Kataoka et al. 2020).

Higher activation was recorded in the CEA‐projecting DP neuronal population in mice exposed to SCS fear conditioning compared to a control group. Interestingly, the highest activation of CEA‐projecting DP cells occurred during WN, with activity increasing concurrently with flight behavior and movement speed. This result was consistent with optogenetic manipulations of DP‐to‐CEA neurons, in which inhibition of such neurons significantly reduced flight in response to WN during the SCS paradigm. Thusly, it is clear that the DP projections to the CEA are crucial in mediating flight responses in high‐intensity threat contexts.

Researchers also found that DP‐to‐CEA circuits especially target specific neurons in the medial subdivision of the CEA (CEM), the main output center that modulates defensive behaviors, thanks to its direct projections to the flight‐mediating periaqueductal gray (a region which has been identified as an effector of defensive behaviors) (Borkar et al. 2024; Fanselow 1991).

In summation, these findings frame the CEA as having an important role in orchestrating conditioned flight as well as other defensive behaviors, thanks to the integration of appropriate context‐driven stimuli (Fadok et al. 2017; Totty et al. 2021). The prefrontal cortex (PFC) has also been identified as having a major role in executive control over defensive responses, exerting direct top‐down control over the CEA. This aligns with previous findings from signaled active avoidance (AA) paradigms using rat models, which demonstrated that a subregion of the PFC, the infralimbic cortex (ilPFC), is involved in regulating defensive behaviors (Moscarello and LeDoux 2013). In AA paradigms, subjects learn to prevent an aversive outcome by executing a specific behavior in response to a conditioned cue. What researchers found was that the ilPFC functions as an inhibitor of CEA‐mediated defensive responses, leading to the suppression of freezing (Moscarello and LeDoux 2013).

Taken together, such evidence may aid in elucidating the neural dysfunctions present in mental illnesses such as PTSD, generalized anxiety disorder (GAD), and panic disorder, given that abnormalities in neural activation of the PFC have been observed in patients suffering from these disorders (Borkar et al. 2024; Tromp et al. 2012). Furthermore, these psychiatric disorders are characterized by chronic dysregulations in the mechanisms that mediate reflexive survival behaviors, such as flight. In the case of GAD, research has shown that affected individuals exhibit reduced autonomic flexibility, marked by chronically elevated sympathetic activity and diminished variability in physiological responses to stress (Friedman and Thayer 1998; Hoehn Saric et al. 1989; Thayer et al. 1996). This autonomic rigidity, reflected in reduced electrodermal activity and slower habituation to repeated stimuli, is thought to impair the ability to generate rapid, high‐energy responses necessary for effective flight behaviors in threatening situations (Hoehn Saric et al. 1989; Newman et al. 2013).

Similarly, PTSD is associated with chronic hyperactivity of the sympathetic nervous system, evidenced by increased heart rate, blood pressure, and skin conductance, among other psychophysiological indicators (Sherin and Nemeroff 2011). However, a subset of individuals with PTSD—referred to as physiological non‐responders—display hypoarousal, reduced affective and physiological responses, and altered activity in fronto‐limbic brain regions, with dissociation playing a major role in these symptoms (American Psychiatric Association 2013). Some researchers suggest that dissociation itself functions as a defensive mechanism against trauma‐related stimuli, leading to a blunted psychophysiological response (Beutler et al. 2022).

## 5 Flight as a Non‐Associative Response

A recent study that replicated Fadok et al.’s 2017 SCS fear conditioning experiments, proposed novel alternative explanations to the behaviors observed (Trott et al. 2022). The researchers mentioned similarities between the recorded flight episodes and the startle response, a typical unconditioned response to a loud noise, which decreases with repeated presentations of the same noise and can be potentiated thanks to a cue or a fear‐inducing context (Davis 1980; Trott et al. 2022). Although the noise used in the experiments was less intense than those normally used in acoustic startle studies, unconditional responses elicited by noise were still induced thanks to the fearful context that potentiated them. Additionally, Trott et al. stated that the neuroanatomical data underlying flight, reported in previous studies, coincided with evidence surrounding the startle response. Specifically, the previously mentioned bed nucleus of the stria terminalis, described by Totty et al., an important mediator for fear potentiated startle, and the CRF expressing cells found by Fadok et al. which have also been recognized as factors that influence startle (Campeau and Davis 1995; Fadok et al. 2017; Lee and Davis 1997; Totty et al. 2021).

Most importantly, the same study proposed a new rule that explains the rapid behavior switch from freezing to flight, reported in fear conditioning studies. The rule specifies that when an animal is in a state of fear, and therefore enacts a post‐encounter defense such as freezing, a sudden stimulus change can enable a circa‐strike defense that is proportional to the stimulus’ intensity and novelty (Trott et al. 2022). Therefore, a novel stimulus, like white noise, can alter the initial post‐encounter state and cause more panic‐like defensive behaviors such as flight. Previous experiments utilized the same context for training and testing, enabling the subjects to readily enter a state of post‐encounter defense thanks to the developed contextual fear. Thusly, the white noise stimuli were especially effective in disrupting freezing and causing panic‐like flight. The same study reported that most of the activity bursts were recorded during the onset of the white noise stimulus and that stronger footshocks triggered longer activity bursts. The timing of the active responses prompted the researchers to consider them as possible alpha responses: unconditional responses to a conditional stimulus that occur at the onset of the CS (Hull 2007; Trott et al. 2022). Since almost all flight‐like activity recorded in their study occurred at the onset of the CS, with almost no US anticipatory behavior, the activity bursts should then be categorized as unconditional noise‐elicited responses to a CS, and not conditioned responses (Trott et al. 2022).

Regarding Fadok and colleagues’ 2017 findings, Trott et al. (2022) suggested that the flight responses recorded weren't necessarily conditioned responses (CR) simply because they increased over trials during acquisition with CS‐US pairings and decreased during extinction with just the CS being presented. Such behavioral properties could also derive from non‐associative processes such as sensitization and habituation. To back their claim, they performed a pseudo‐conditioning control experiment in which only shocks were administered, thus conditioning fear to the context. Interestingly, after presenting the white noise stimulus for the first time it elicited strong activity bursts, that later decreased after repeated presentations of the same noise. All behavioral patterns frame flight as a non‐associative response (Trott et al. 2022).

Finally, with respect to the type of stimulus, the study reported that both white noise and pure tones were able to induce flight, although noise triggered a greater number of high‐velocity movements. A similar result was obtained in a 2023 study by Furuyama et al. (Furuyama et al. 2023), who conducted modified fear conditioning experiments on male mice which, unlike previously mentioned studies, didn't use neither SCS nor white noise. The CS used was an 8 kHz continuous tone that lasted 20 seconds, and the US was a footshock delivered immediately after the tone. After repeated contextual fear conditioning sessions, the researchers were able to consistently trigger flight responses (recorded as an increase in jumps and movement) using a salient CS. By manipulating the volume of the CS, they also found that tones at lower volumes (75 dB) triggered less flight compared to louder ones (95 dB). Furthermore, the expression of flight seemed to be influenced by the number of conditioning sessions, CS‐US pairings and by spacing conditioning trials across multiple days. Flight behaviors observed during conditioning sessions appeared during the entire pure tone presentation, they emerged following contextual conditioning without any salient stimuli present, and finally they weren't triggered by loud noises presented during habituation (Furuyama et al. 2023).

## 6 Associative and Non‐Associative Learning

In response to the discussion on the non‐associative factors of flight, Le and colleagues performed new Pavlovian fear conditioning experiments with the aim of elucidating the effects of associative and non‐associative mechanisms on defensive behaviors (Le et al. 2023). To do so they divided their mice subjects into three experimental groups: one that received US and SCS in an unpaired and non‐predictive way (referred to as UN in the study), a group that only received shocks during conditioning (SO) and a paired SCS‐shock group (PA). The PA group was designed to test whether flight behavior is learned through association, while the UN and SO groups controlled for non‐associative factors such as stimulus salience and sensitization, which Trott et al. argued could explain flight (Le et al. 2023). After the conditioning phase, all groups were subjected to two extinction sessions with only SCS presentations.

Le et al. (2023) found that the contingency between the SCS and the US during conditioning was fundamental for the expression and extinction of stimulus‐induced defensive behaviors. Greater changes in defensive behavior during WN and tone were recorded in mice that underwent paired SCS‐US conditioning, showing significant freezing to the tone, and jumping and darting during the entirety of WN presentations. Furthermore, PA mice showed an increase in freezing from pre‐SCS to tone periods, while the UN mice's freezing in response to tones wasn't greater than their contextual freezing: contrasting results that evidence how the PA group placed associative value on the tones. PA mice also exhibited consistent jumping and darting during WN, while the UN group didn't. From this data, the authors recognized that jumping and darting in PA mice were associative learned defensive responses and were not caused by stimulus salience. If flight were purely a non‐associative response, then all groups should have exhibited similar jumping and darting behavior when exposed to the SCS. However, mice in the UN group didn't associate WN to incoming threat, so, instead of showing “appropriate” conditioned defensive responses, their behavior reflected standard locomotion and freezing caused by stimulus salience (Le et al. 2023).

After analyzing the behavioral ethograms of each group during the extinction sessions, the researchers found that the PA group, having learned that WN no longer indicated an incoming threat, decreased their activity during WN and instead enacted more anticipatory freezing and darting. In contrast, UN and SO groups didn't display any freezing during WN periods and maintained a high activity index throughout the extinction sessions, responding similarly to stressed mice to an unfamiliar WN. In the first trials of extinction, mice in the PA group enacted more jumping behaviors, during the entirety of the WN stimulus, compared to the other groups. By the end of the first extinction session, PA mice showed less jumping and, along with the SO group, more darting; a result that indicates jumping as an associative defense that is replaced with darting as the threat becomes less imminent (Le et al. 2023).

Non‐associative factors did elicit some stress‐induced behaviors, however the researchers insisted that they weren't significant contributors to intense cue‐evoked defenses (Le et al. 2023). For example, tail rattling was observed during SCS presentations in early extinction trials mostly among UN and SO mice during tone presentations, with UN mice showing more tail rattling during WN than PA mice. Le et al. interpreted this data as proof that tail rattling to the conditioned stimulus is a non‐associative response that occurs in stressful scenarios where danger is uncertain but anticipated, and it decreases when the stimulus signals an imminent threat.

## 7 Alternative Explanations for White Noise Effects

Although there are more ecologically valid ways to study flight behavior, it is particularly interesting that this response is linked to a seemingly “harmless” feature of the stimulus—its white noise characteristics, which are common in dynamic systems. The brain itself is a highly dynamic system, making the study of responses to white noise a unique opportunity to understand how the brain processes stimuli, especially those that trigger flight behavior.

Indeed, after uncovering how WN stimuli can activate different neuronal populations, as reported in the previous paragraphs, it is now important to explore alternative explanations for the phenomena described above. Namely, through what different paths does the white noise stimulus reach the amygdala? And to what different effects can such pathways lead?

One example is the hypothesis that WN stimuli, such as most anthropogenic noises, can be disruptive and cause difficulties in perceiving other sounds (due to their wide frequency range), causing animals to miss important auditory information from their surroundings that might warn them of imminent danger (Kelligrew et al. 2021). Alternatively, according to the distracted prey hypothesis (Chan et al. 2010), WN can simply be a source of distraction, forcing prey animals to divide their attention and be less ready to engage in risk assessment behaviors. More generally, different studies have observed that WN exposures cause animals to be more vulnerable to threats, be it by slowing animals’ reaction time or by delaying their flight response (which contradicts SCS findings) (Kelligrew et al. 2021).

A 2021 study by Kelligrew et al. (2021) investigated the effects of WN exposures on blue‐tailed skinks *Emoia impar* by measuring their behavioral response as well as their flight initiation distance (FID). What the researchers found was that white noise had no direct effect on FID, thus excluding the possibility that it could distract the subjects and worsens their risk assessment behavior by fleeing later than expected. However, WN exposures did heighten the skink's vigilance, by increasing their looking and locomotion rates. Furthermore, subjects with higher responsivity to white noise (showing greater looking rates) had greater FIDs and fled earlier. Finally, the researchers noted that the observed heightened responsivity in skinks during WN exposures supported previous claims suggesting prey animals perceive white noise as a threatening stimulus, causing them to increase their vigilance and antipredator behavior (Kelligrew et al. 2021).

A 2014 study on white‐crowned sparrows (*Zonotrichia leucophrys*) by Blesdoe and Blumstein led to similar conclusions. Here the researchers exposed the sparrows to different synthesized sounds to uncover the types of reactions elicited by non‐linear acoustic stimuli, i.e., desynchronized vibrations created by a sound production system (for example, white noise). Their experimental paradigm included four different sound conditions: one control and three experimental stimuli. The control stimulus was composed of a 0.5 s pure tone at 3 kHz, while all three experimental stimuli started off with 0.4 s of a 3‐kHz pure tone and ended with either a frequency jump up an octave (to a 5‐kHz pure tone), a frequency jump down an octave (to a 1.5‐kHz pure tone), or white noise with a range of 1–5 kHz. After each stimulus the researchers quantified the degree of relaxed behavior expressed by the sparrows by observing the amount of time spent performing typical relaxed behavior during baseline recordings and comparing them to the experimental conditions (e.g., foraging, vocalizing, and walking).

Once again, white noise exposures seemed to cause higher vigilance, expressed by significantly less relaxation, compared to pure tones. Furthermore, downward frequency jump exposures elicited less relaxation than pure tones, while the sparrows did not show significant decreases in relaxation after upward frequency jumps. These results confirmed the study's hypothesis of nonlinear sounds causing the sparrows to show higher arousal (Blesdoe and Blumstein 2014).

The researchers also noted how their findings go hand in hand with previous explanations of naturally occurring nonlinear sounds, one of them being the unpredictability hypothesis. This adaptive hypothesis posits that naturally occurring nonlinear sounds (such as baby cries or alarm calls) are fundamentally unpredictable, thus making animals less likely to be easily habituated to them, causing them to stand out and be much more salient than other sounds. Finally, it has been stated that sounds that aren't easy to habituate to also tend to elicit more heightened behaviors. Although, Blesdoe and Blumstein do mention how this might be due to the general pattern where nonlinear sounds are often produced under highly stressful situations.

Another relevant finding linking WN exposures to increased vigilance was uncovered by a 2016 study from Baijot et al. (2016)that tested the assumption that children suffering from attention‐deficit/hyperactivity disorder (ADHD) would benefit from WN stimuli in an attention and inhibition task (a visual cued Go/Nogo task). This hypothesis was based on the moderate brain arousal (MBA) model, which states that random noise present in the environment can produce internal noise in the neural system, thus compensating the reduced background neural activity in ADHD patients associated with a less active dopaminergic system (Baijot et al. 2016). Researchers in the study based their predictions also on the optimal stimulation theory, which posits that individuals all have a predetermined optimal level of arousal allowing them to properly function cognitively. Similarly, to the MBA model, optimal stimulation theory suggests that children suffering from ADHD have a lower biologically determined level of arousal, causing them to perform poorly in normal conditions, and that by adding environmental noise, in the form of extra‐tasks, parallel to the main task, ADHD children would be able to reach their high‐stimulation threshold and perform better. The researchers compared the performance of children with and without ADHD in the Go/Nogo task while being exposed to white noise or no noise. Meanwhile, neurophysiological and physiological measures were taken: the mean amplitudes of P300 (an event‐related potential, ERP, component often used as a marker in ADHD) and spontaneous eye blink rates (used as indirect markers of dopamine production in the striatum). What they found was that WN exposures benefitted the cognitive performance of children with ADHD during the task, in accordance with both the optimal stimulation theory and the MBA model. However, the positive impacts of WN was only limited to the omissions, while the number of false alarms, reaction time, and reaction time variability was still higher in children with ADHD compared to typically developing children in both WN and no‐noise conditions. The researchers interpreted the effect of WN as exclusively increasing vigilance in ADHD subjects. At an electrophysiological level, subjects that benefitted from white noise had marginally larger mean amplitude of the P300 ERPs during Go trials, which are associated with orienting and attentional processes, such as vigilance (Baijot et al. 2016).

Finally, it is important to mention the more physical properties of white noise and their effects on our sensory system. It has been well documented that broadband noise can aid neurons in detecting weak signals via a mechanism known as stochastic resonance (SR) (Nozaki et al. 1999). More specifically, SR occurs when a weak, undetectable, sub‐threshold signal becomes detectable thanks to the presence of noise. Noise allows the stimulus to be encoded by the sequence of threshold crossings that commonly appear at the peaks of the signal, which are closest to the threshold. Thus, the added noise is able to sample the subthreshold stimulus’ amplitude at a given time, represented by the sequence of threshold crossings. Additionally, there is an optimal level of noise that maximizes the amount of information transferred (Moss 2004).

Previous studies proved how SR occurs in animals, evidencing how adding external noise can cause animals to perceive enhanced information in their peripheral sensory system, allowing them to use this information for feeding or avoiding predators. Evidence from psychophysics studies suggests that SR is also present in human hearing. For example, people with cochlear or brainstem implants were able to better detect pure tones with the addition of a broadband noise (Moss 2004).

White noise in particular has been widely used in experimental settings to obtain SR‐type effects. For instance, SR has been reproduced in rat sensory neurons, specifically cutaneous afferents in the skin, using white noise (Nozaki et al. 1999). However, we believe that it would be insightful to study neural SR‐type effects using different colored noises (noises with different power spectrums), such as pink or brown noise, which each have specific properties that have not been explored in such experiments.

Taken together, this evidence supports the need for a more in‐depth investigation of the neurophysiological responses to noisy stimuli, in particular their differential effect on the two branches of the autonomous nervous system, sympathetic and parasympathetic, leading to the selection of behavioral defensive responses.

## 8 Discussion

Today, the flight response mechanisms and their underlying neural circuits are still unclear, although it is evident that novel therapeutic interventions would reap great benefits from elucidating said aspects. Taken together, the aforementioned SCS paradigms have proven to be major tools in understanding flight, demonstrating clear advantages in reproducing and analyzing circa‐strike responses, rarely studied in classical Pavlovian fear conditioning experiments. Moreover, said paradigms enabled researchers to uncover important neuronal mechanisms involved in fear and anxiety‐related mental disorders, such as post‐traumatic stress disorder and generalized anxiety disorder (Borkar et al. 2024).

However, it goes without saying that the SCS paradigm could be modified and adapted to the study of different aspects of defensive response mechanisms, providing a more comprehensive understanding of their dynamics. Here we propose possible aspects that could be incorporated within the SCS paradigm by future studies.

One element of defensive responses that could be analyzed, using the SCS paradigm, is motivation. In particular, the introduction of a possible escape route from the area associated with danger in the experimental enclosure, could be used in order to produce stronger escape behaviors and to study the animal's motivation to escape from danger. The inclusion of this variable in the paradigm is supported by studies that have found escape routes to play an important role in influencing the flight response. This was clearly portrayed in a 1997 study by D. Caroline Blanchard in which wild rats were placed in a large oval‐shaped runway that was big enough to allow unlimited forward movement (Blanchard 1997). Afterwards, the experimenter would gradually approach the enclosure at a certain speed. Thus, a flight response was elicited in the animals with a mean percentage of avoidance of 97.3 %. The situation drastically changed when the enclosure was transformed into an inescapable straight corridor, thanks to the closing of a door. In this scenario, flight responses were virtually replaced by freezing, a response present at an average of 100% (Blanchard 1997).

This could be implemented within the SCS paradigm, for example, by limiting the area of the electrified grid of the enclosure that delivers footshocks (perceived as the danger stimuli) to just half of the grid. To avoid having the rats quickly learn to remain on the non‐shock side, the electrified half of the grid could be randomized between trials. By doing so, the animals would have a viable escape route from the “dangerous area” (the electrified half of the grid) to the “safe area” (the other non‐electrified half), granting a more directionally motivated flight response. Observers would then be able to study and quantify the animals’ motivation to escape from the footshocks by analyzing their escape speed.

Finally, electrifying only half of the grid could also be used as a countermeasure for the induction of learned helplessness in the animals, a state that occurs following exposure to repeated, inescapable stressful stimuli and in which the subjects feel passive and unable to control the outcomes of the event, virtually eliminating their attempts to flee or fight back (Maier and Seligman 1976).

Apart from offering important insight on directionally motivated flight responses, this type of modified SCS paradigm could be useful in analyzing depressive‐like behavior, since motivation, more specifically an impairment in motivational processes, represents a fundamental aspect in depression (Grahek et al. 2019). For instance, this new paradigm could be tested in place of or parallelly to other behavioral protocols, such as the forced swim test (FST), to quantify and characterize depressive‐like behaviors induced by chronic mild stress (Lamanna et al. 2022) or increased motivation induced by acute mild stressors (Lamanna et al. 2021). Indeed, the FST is one of the most commonly used rodent behavioral tests for the study of depressive‐like behavior and antidepressant efficacy (Can et al. 2011; Yankelevitch‐Yahav et al. 2015). In FST experiments, mice or rats are placed in inescapable transparent containers filled with water. Observers then measure the animals’ escape‐like movements, such as swimming and sometimes climbing, until the subject eventually loses hope in trying to escape and becomes immobile. The latter described behavior is denoted as behavioral despair and seems to be an important animal model for studying depression (Bogdanova et al. 2013). However, for some time, researchers have identified several limitations and issues within the FST paradigm. For example, the immobility shown by the subjects during the previously mentioned behavioral despair phase, has an unclear meaning regarding the type of depressive symptoms it reflects. Interestingly, it has been hypothesized that the animals choose to be immobile during the test to preserve energy with hopes of using it for a possible future escape (Yankelevitch‐Yahav et al. 2015). Additionally, some researchers posit the existence of a so‐called learned immobility, which is a learned process in which the subjects learn that the easiest solution is to wait passively until they are removed from the water (De Pablo et al. 1989).

Considering these limitations, the use of a novel paradigm for the study of depressive‐like behaviors in rodents presents clear advantages in providing new insight on the matter. This paradigm will induce subjects to be motivated to move away from the danger (the electrified half of the enclosure), and an inability to do so might more easily be ascribed to an impairment in motivational processes.

Finally, two other important aspects that could be implemented in the SCS paradigm are threat distance and threat approach speed. It has been by now well established that defensive distance, i.e., the distance between prey and predator, strongly influences defensive behavior (Blanchard and Blanchard 2008). Specifically, the more defensive distance decreases, and the probability of direct contact with the predator increases, the more circa‐strike‐like the prey's antipredator behavior becomes, with flight elicited when predators are very close. However, recently it has been shown that flight is also mediated by the predator's approach speed (Cooper 2006). Namely, FID (i.e., the distance between the prey and the predator in the instant that flight begins) is greater in the case of a rapidly approaching predator as opposed to a slowly approaching one.

These flight mediators could also be integrated into the SCS paradigm. A possible way to do this could be via the manipulation of the volume of the white noise period within each trial during the conditioning phase.

This possible change in protocol is supported by the results from Hersman et al.’s (2020)previously mentioned 2020 study, which demonstrated how mice perceived stimuli at higher sound pressure levels (SPL) as more imminent by conducting an “SPL step test” for the pure tones and later the white noise pips of the SCS, within which they would increase the volume of one of the stimuli by a certain amount each trial. However, to our knowledge, an intra‐trial volume increase (i.e., a manipulation of the volume within the single SCS presentation) has never been performed in such behavioral experiments.

By increasing in real time, the volume of the white noise, which tends to be perceived as an imminent threat (Fadok et al. 2017), it seems plausible to be able to simulate an approaching threat, so as to more strongly induce a motivated circa‐strike defensive response. By doing so, one will also have the advantage of simulating a more naturalistic scenario of a moving threat, instead of one fixed at a set distance. Additionally, different conditions characterized by different trends of increasing white noise volumes could be designed in order to reflect a threat approaching at different speeds.

## 9 Conclusions

Most classical fear conditioning studies have solely concentrated on reproducing and analyzing post‐encounter, freeze‐like behaviors. Luckily, the study of circa‐strike responses, such as flight, has become more common within the past decade. This change in focus has uncovered many informative findings on innate defensive responses as well as on the nature of mental disorders, such as anxiety, through numerous translational science experiments. Moreover, the aforementioned SCS paradigms provided groundbreaking evidence supporting the ability to condition flight, which appears to challenge fundamental assumptions of widely accepted theories on defensive behavior and suggests that these theories should be revised to better incorporate this evidence. Additionally, this novel approach seems particularly promising in that it could allow researchers to study the complex behavioral switches that occur between fear and other defensive modes, like panic or anxiety, as well as their underlying neuronal processes.

This new trend in behavioral science has also introduced a significant methodological advantage. That is, researchers have begun to give more attention towards the different properties of the audio stimuli used in their experiments, i.e., pure tones and white noise. And by doing so, they were able to isolate with greater clarity the effects caused by the stimuli, thus more directly manipulating behavior and neuronal activity. Finally, knowing the properties of the stimuli used in the experiment aids in distinguishing the effects caused directly by the stimuli from those caused simply by association.

## Author Contributions

**Sebastiano Francesco Matarazzo di Licosa**: investigation, writing–review and editing, writing–original draft. **Andrea Stefano Moro**: writing–original draft, writing–review and editing. **Mattia Ferro**: writing–review and editing. **Antonio Malgaroli**: writing–review and editing. **Jacopo Lamanna**: conceptualization; writing–review and editing, writing–original draft.

### Peer Review

The peer review history for this article is available at https://publons.com/publon/10.1002/brb3.70561.

## Data Availability

Data sharing is not applicable to this article as no new data were created or analyzed in this study.
